# Development of Composite Edible Coating from Gelatin-Pectin Incorporated Garlic Essential Oil on Physicochemical Characteristics of Red Chili (*Capsicum annnum* L.)

**DOI:** 10.3390/gels9010049

**Published:** 2023-01-06

**Authors:** Windy Heristika, Andriati Ningrum, Supriyadi, Heli Siti Helimatul Munawaroh, Pau Loke Show

**Affiliations:** 1 Department of Food and Agricultural Product Technology, Faculty of Agricultural Technology, Universitas Gadjah Mada, Flora Street No. 1, Bulaksumur, Yogyakarta 55281, Indonesia; 2 Study Program of Chemistry, Department of Chemistry Education, Faculty of Mathematics and Science Education, Universitas Pendidikan Indonesia, Bandung 40154, Indonesia; 3 Department of Chemical and Environmental Engineering, University of Nottingham Malaysia, Jalan Broga, Semenyih 43500, Selangor Darul Ehsan, Malaysia

**Keywords:** edible coating, fish gelatin, red chili, pectin, garlic essential oil

## Abstract

Red chili is a climacteric fruit that still undergoes respiration after harvest. During storage, it is susceptible to mechanical, physical, and physiological damage and decay incidence, therefore a method is needed to protect it so that the quality losses can be minimized. One way this can be achieved is by applying edible coatings that can be made from hydrocolloids, lipids, or composites of both, in addition to antimicrobial agents that can also be added to inhibit microbial growth. In this study, we detail the application of an edible coating made of gelatin composite from tilapia fish skin, which has a transparent color and good barrier properties against O_2_, CO_2_, and lipids. To increase its physicochemical and functional qualities, it must be modified by adding composite elements such as pectin as well as hydrophobic ingredients such as garlic essential oil. This study was conducted to determine the effect of a gelatin–pectin composite edible coating (75:25, 50:50, 25:75), which was incorporated with garlic essential oil (2% and 3%) on the physicochemical properties of red chili at room temperature (±29 °C), RH ± 69%) for 14 days. The best treatment was the 50–50% pectin–gelatin composite, which was incorporated with garlic essential oil with a concentration of 2 and 3%. This treatment provided a protective effect against changes in several physicochemical properties: inhibiting weight loss of 36.36 and 37.03%, softening of texture by 0.547 and 0.539 kg/84 mm^2^, maintaining acidity of 0.0087 and 0.0081%, maintaining vitamin C content of 2.237 and 2.349 mg/gr, anti-oxidant activity (IC_50_) 546.587 and 524.907; it also provided a protective effect on chili colors changing to red, and retains better total dissolved solid values.

## 1. Introduction

Chili is a fruit plant that is very common in everyday life, and in Indonesia, almost everyone uses it in food and as a spice in daily cooking [1]. Chili can be harvested when it is still green and when it is ripe [1]. Because chili is a climacteric fruit, the physicochemical properties of chili can be changed during the ripening stage due to respiration, production of ethylene, and other physiological reactions. Because of their color, pungency, flavor, and perfume, chilies (*Capsicum* sp.) are employed as a spice in many national cuisines [2]. A good quality chili or pepper has several characteristics such as firmness, fresh calyx and pedicel, and lack of bruises, abrasions, and disease. Shrivel and wilting both have a significant impact on the visual quality of chilies [3]. Damage to chili during storage, especially at room temperature, includes physical damage such as injury, loss of weight, decreased water content, firmness, freshness, and vitamin C content [3]. 

Chili can be marketed in modern markets or traditional markets. In modern markets, chilies are occasionally packaged in plastic wrap and stored at a cooler temperature. This is in contrast to sales in traditional markets, in which chilies are usually stacked in an open room. When the chili arrives in the hands of consumers, it is sometimes only stored at room temperature at home. Therefore, it is necessary to find a method to be able to extend its shelf life, especially at room temperature [4].

Currently, the use of natural preservatives is increasingly in demand and has garnered special attraction. One method that can be applied to maintain quality and extend the shelf life of fruits and vegetables is to provide an edible coating [5,6,7,8]. Chili with a thin edible coating can be consumed without the need to be separated or thrown away because the edible coating is not dangerous when ingested in the body [9,10].

Edible coatings can be made from hydrocolloids, lipids, or composites of the two. In addition, antimicrobial agents can also be added to inhibit microbial growth of the product being coated [11,12,13,14]. Another consideration that is also important in choosing this coating solution material is raw materials that are sustainable, environmentally friendly practical, and economical [15,16,17]. The use of materials according to these criteria will certainly increase stability and protection during the product’s shelf life and is expected to reduce total operational costs while reducing waste generated [13,14,18,19]. One of the most widely applied biopolymers for various types of food is gelatin. Gelatin has good mechanical properties, good optical properties, and a barrier effect on the gas flow [20,21]. An alternative source of gelatin that can be utilized is gelatin from fish industry waste [20,22]. The use of by-products or agro-industrial and aquatic waste such as tilapia fish skin by-products will add economic value and have a positive impact from an environmental and social perspective [21,23]. Tilapia skin gelatin is a species of warm water fish with a greater bloom value than cold water fish with gelling and melting values similar to mammalian gelatin, making it suitable for use as an edible coating [10].

The use of gelatin has a limitation—namely, it has a fairly high value of water vapor permeability (WVP) due to the hydrophilic nature of gelatin; therefore, it is necessary to improve this property [24,25]. One of the ways to improve the properties of gelatin is to composite it with polysaccharides such as pectin, which can correct these deficiencies. Pectin has good mechanical properties and is a good barrier to oxygen and oil [26].

The usage of gelatin in food has been widely employed, although edible coatings have been widely used on chili peppers themselves, including coating with chitosan, which has been proven to retain quality and decrease damage to green pepper [7]. In addition to composites, we can also improve the mechanical and also functional properties of edible coatings by adding antimicrobial agents such as essential oils to slow down spoilage. Essential oils that have been widely used, including rosemary in gelatin from fish, have been shown to reduce WVP by up to 54% because essential oils are hydrophobic so they can improve the hydrophilic properties of gelatin. In addition, garlic essential oil is an antifungal agent, and mycotoxins in corn and edible films are made of protein [27].

Several studies on the use of edible coatings with composite materials on fruits and vegetables—especially chili commodities—have been carried out [28]. The application of chitosan–gelatin composite material as an edible coating on post-harvest red bell pepper demonstrated excellent firmness retention compared to single coatings (noncomposite coating) and untreated fruit [10]. Few reports are available on the use of the edible coating gelatin–pectin composite with incorporated garlic essential oil in red chili to extend the period of usability of red chili; however, to the best of our knowledge, no data is yet available on the combined influence of this composite, or on expanding the shelf-life and maintaining red chili quality traits. As a result, the purpose of this experiment was to investigate the effects of the application of the edible coating gelatin–pectin composite with incorporated garlic essential oil on the shelf-life, weight loss, physicochemical parameters (such as color, firmness, vitamin C, titrable acidity) of red chili, and disease incidence during their postharvest storage at room temperature.

## 2. Results and Discussion

### 2.1. FTIR

**Figure 1:**
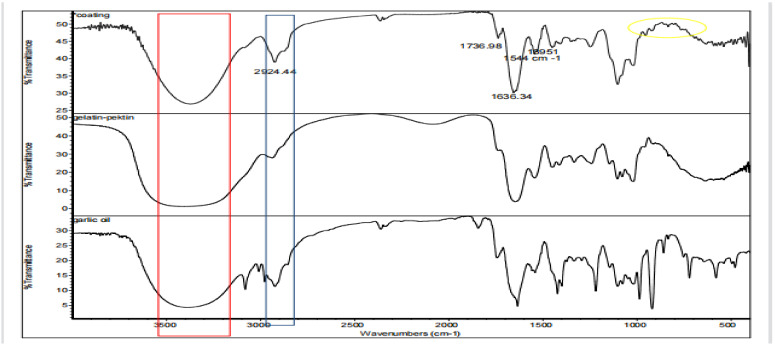
FTIR of an edible coating of Tilapia gelatin composite gelatin–pectin with incorporated garlic essential oil.

The FTIR (Fourier Transform Infrared) spectra of a gelatin–pectin composite coating solution containing garlic essential oil and a gelatin–pectin coating solution without garlic oil are shown in Figure 1. In general, both coating solutions showed similar main peaks due to the predominant gelatin–pectin composition compared to the essential oil concentration.

The presence of polyhydroxy chemicals such as flavonoids, nonflavonoids, and saponins is indicated by the existence of a wide peak at wave numbers 3373.49 and 3404.91 cm^−1^ on samples with coating with essential oil and without essential oil, respectively. The coating solution containing the essential oil has a higher hydrophobicity than the coating solution without essential oil, as evidenced by the highest peak amplitude at wave number 2924.44 cm^−1^. This is following the hydrophobic moieties CH, CH_2_, CH_3_ as aromatic compounds. Peak is also seen at wave number 2363 cm^−1^ like the peak that also appears in the spectrum of garlic essential oil. Strong peaks were also found at about 1630 cm^−1^ for coating with essential oils, showing the existence of carbonyl and carboxylic group stretching. The peak at 1395 cm^−1^ reveals the presence of flavonoids, tannins, saponins, and glycosides by indicating the O-H bend of carboxylic acids. The peak at 1036 cm^−1^ indicates the organosulfur group including alliin, allicin, and diallyl disulfide [29].

The peak at approximately 720 cm^−1^ indicates the C-S bond strain in garlic oil. In the standard spectrum of garlic oil, high-intensity peaks appear in the range of 990 cm^−1^ to 900 cm^−1^. These peaks are caused by the =CH_2_ deformation of the vinyl groups found in sulfides and dithiins. This peak was also seen in the coating samples, but it was not as strong as the standard essential oil. Thus, it can be concluded that essential oils can provide a cross-linking effect with the coating solution.

### 2.2. Weight Loss

**Figure 2:**
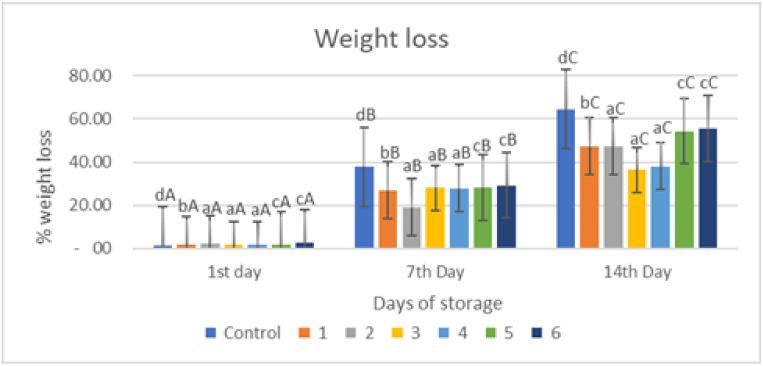
Effect of an edible coating of Tilapia gelatin–pectin composite with incorporated garlic essential oil on weight loss of red chili during storage The value shown is the average of the three experimental replications. Control: Uncoated Sample; 1: Gelatin75%-Pectin25%-Garlic Essential Oil2%; 2: Gelatin75%-Pectin25%-Garlic Essential Oil Oil3%; 3: Gelatin50%-Pectin50%-Garlic Essential Oil Oil2%; 4: Gelatin50%-Pectin50%-Garlic Essential Oil Oil3%; 5: Gelatin25%-Pectin75%-Garlic Essential Oil Oil2%; 6: Gelatin25%-Pectin75%-Garlic Essential Oil Oil3%. ^a–d^ non-capital letters show the difference between the samples (*p* < 0.05). ^A–C^ capital letters show the difference between the day of storage (*p* < 0.05).

Figure 2 indicates that applying an edible gelatin–pectin coating supplemented with garlic essential oil to red chili reduces the percentage of weight loss in red chili held at room temperature for 14 days more efficiently than the uncoated sample (*p* < 0.05). The proportion of weight loss in untreated samples (control) was much greater than in samples coated with an edible coating (Figure 2). Weight loss is commonly attributed entirely to water loss; however, loss of other components may also contribute to this problem. Nonetheless, other than water loss, the contribution from other components is regarded as minimal. This water loss reduces the turgor and hardness of the fruits. It could cause acceleration in the surface depression and deformation of produce. Water loss is associated with several other changes occurring in fruits and can act as a trigger to initiate these changes [30]. The edible coatings can cover the surface layer of the fruit, preventing respiration, transpiration, and syneresis [8,15,16]. This data supports the advantages of putting the edible coating on fresh-cut red chili pieces, owing to the establishment of a polymeric barrier, which has been shown to prevent water loss from fresh-cut samples in other fruits [17,18].

Figure 2 depicts the significant weight decrease of the treated and untreated fruits (*p* < 0.05). This weight reduction in the items might be attributed to moisture loss, transpiration, and respiration, which gave the weight loss of the red chili. Water loss in the fruit occurs mostly through the fruit cuticle, the fruit’s physical properties, or both. The loss of a carbon atom in each cycle of respiration may result in weight loss as well. A similar result has been made about bell peppers and green chilies. As a result, an appropriate concentration of applications of edible coating might prevent weight loss [17,18].

Overall, based on Figure 2, the percentage of weight loss increased with storage time, the percentage of weight loss in the control sample on the 1st, 7th and 14th observation days was 1.12, 37.76, and 64.71%, respectively, while in the coated samples the highest weight loss was in samples 5 and 6 at 54.48 and 55.57%, respectively, on the 14th day of storage, and the lowest weight loss was in samples 3 and 4 at 36.36 and 38.03%, respectively, on the 14th day of storage. Based on Figure 2, treatment and storage time had a significant effect (*p* < 0.05) on weight loss.

### 2.3. Total Vitamin C

**Figure 3:**
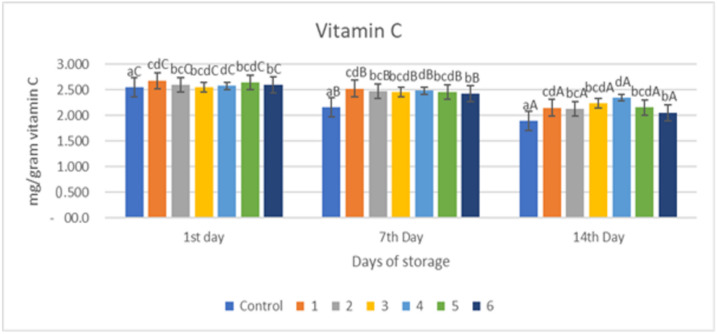
Total Vitamin C of red chili during storage. ^a–d^ non-capital letters show the difference between the sample (*p* < 0.05). ^A–C^ capital letters show the difference between the day of storage (*p* < 0.05). Control: Uncoated Sample; 1: Gelatin75%-Pectin25%-Garlic Essential Oil2%; 2: Gelatin75%-Pectin25%-Garlic Essential Oil Oil3%; 3: Gelatin50%-Pectin50%-Garlic Essential Oil Oil2%; 4: Gelatin50%-Pectin50%-Garlic Essential Oil Oil3%; 5: Gelatin25%-Pectin75%-Garlic Essential Oil Oil2%; 6: Gelatin25%-Pectin75%-Garlic Essential Oil Oil3%.

Figure 3 shows the results of an evaluation of the vitamin C levels in red chili with an edible coating of a gelatin composite supplemented with garlic essential oil. Figure 3 shows that there was a significant reduction in the control sample and the sample with the edible coating treated with the addition of essential oil (*p* < 0.05). The decrease in vitamin C levels in red chili during storage was caused by the oxidation process. Vitamin C oxidizes rapidly to L-dehydroascorbic acid and then to L-dicotigulonic acid [19]. Changes in vitamin C content are caused by the unstable nature of vitamin C, which is easily oxidized when exposed to oxygen. Vitamin C contains a hydroxyl functional group (OH) that is highly reactive in the presence of an oxidizing hydroxyl group and will be oxidized to a carbonyl group. This is in line with research conducted that showed that there was a decrease in vitamin C levels in samples during storage [31]. Ascorbic acid (vitamin C) is a hydrophilic antioxidant that scavenges damaging harmful additional ROS and free radicals. In the present research, in all treatments, the ascorbic acid content declined continuously throughout the storage period, and on day 14 of storage, its content in the control samples (untreated) decreased higher than in the coated sample. The use of an edible coating gelatin–pectin composite containing garlic essential oil decreased the loss of ascorbic acid in red chili.

The decline in vitamin C in samples without treatment (control) was substantially greater (*p* < 0.05) than in samples treated with an edible coating (Figure 3). This is because the application of an edible coating can inhibit the diffusion of O_2_ into the fruit tissue. Therefore, the oxidation reaction that causes damage to vitamin C can also be inhibited. Meanwhile, in untreated samples (control), the diffusion of O_2_ was not inhibited as a result, and vitamin C degradation continued. Our findings demonstrated that the edible coating gelatin–pectin composite incorporated with garlic essential oil had a substantial effect on retaining the ascorbic acid content of red chili after 14 days of storage at room temperature. The capacity of the edible coating composite film to improve the inner atmosphere status and postpone the ripening process of red chili was linked to the gradual drop in ascorbic acid level in coated samples.

Naturally, ascorbate oxidase is the primary enzyme responsible for the degradation of ascorbic acid. Ascorbate oxidase levels increase under stress. Ascorbic acid oxidation can result in a loss of nutritional value. Essential oils have antioxidant properties and can help to minimize oxidative stress. As a result, it appears that an edible coating composite of gelatin–pectin incorporated with garlic essential oil improves the antioxidant activity of the investigated oil over time by protecting it from degradation by oxygen, light, and temperature, resulting in less ascorbic acid degradation in the treated sample.

The value of vitamin C from the chili samples treated with coating and the control samples during the storage period can be seen in Figure 3. From Figure 3, it can be seen that there was a decrease in the content of vitamin C (*p* < 0.05) during the storage period in all samples. Samples with a composition of gelatin:pectin 50:50 (samples 3 and 4) were able to maintain the highest vitamin C content, namely 2.237 and 2.349 mg/g; as well as for other samples treated, the greater the concentration of essential oil given, the better it was in maintaining the vitamin C content. The uncoated control sample had the lowest vitamin C content on day 14 of storage, namely 1.898 mg/g, and was significantly different (*p* < 0.05) compared to all samples treated on all observation days. 

### 2.4. Firmness

**Figure 4:**
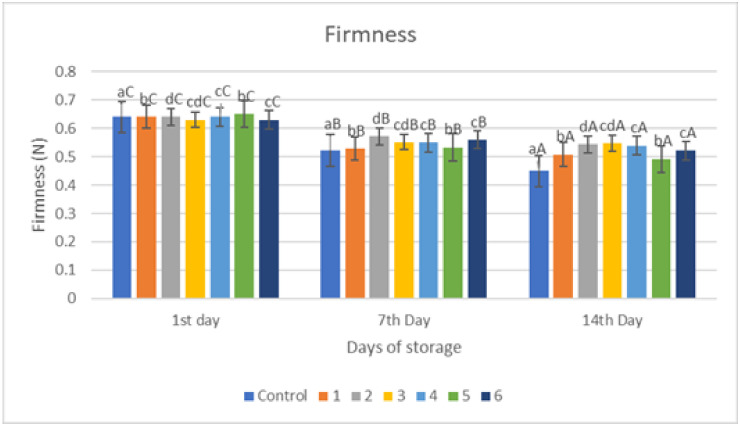
Firmness of red chili during storage. ^a–d^ non-capital letters show the difference between the sample (*p* < 0.05). ^A–C^ capital letters show the difference between the day of storage (*p* < 0.05). Control: Uncoated Sample; 1: Gelatin75%-Pectin25%-Garlic Essential Oil2%; 2: Gelatin75%-Pectin25%-Garlic Essential Oil Oil3%; 3: Gelatin50%-Pectin50%-Garlic Essential Oil Oil2%; 4: Gelatin50%-Pectin50%-Garlic Essential Oil Oil3%; 5: Gelatin25%-Pectin75%-Garlic Essential Oil Oil2%; 6: Gelatin25%-Pectin75%-Garlic Essential Oil Oil3%.

The results of the firmness analysis of red chilies coated with an edible coating of a gelatin–pectin composite incorporated with garlic essential oil can be seen in Figure 4. Firmness is a very important parameter in determining the quality of chili that can be accepted by consumers, and this hardness value will decrease linearly during the storage period even though the samples have been treated. The F_max_ value indicates the hardness level of the sample; higher F_max_ values indicate that the sample tested has a harder texture. Figure 4 shows similar results in that the hardness value of all samples decreased with increasing storage time. The largest significant decrease in hardness value (*p* < 0.05) was in the control sample on all days of storage, from 0.64 kg/84 mm^2^ on the first day to 0.449 kg/84 mm^2^ on the 14th day. Meanwhile, the hardness value for each sample differed significantly from day to day. The best hardness results during storage occurred in samples with coating 3, namely from 0.63 kg/84 mm^2^ on the first day to 0.547 kg/84 mm^2^ on the 14th day. Therefore, based on the results coating 3 gives the best results.

The results of the firmness of red chili during storage revealed that the gelatin–pectin composite edible coating mixed with garlic essential oil greatly delayed the decline of the firmness in the coated sample when compared to red chili control samples. The firmness of the room control samples decreased more throughout the 14-day storage period. This might be attributed to an increased rate of transpiration by the fruit tissue, which leads to a loss of cell turgor and, as a result, tissue stiffness. Firmness retention was greater in treated samples compared to controls under both storage conditions [17,18]. The results of this investigation demonstrated that a gelatin–pectin composite edible coating infused with garlic essential oil considerably delayed firmness loss.

Several mechanisms—including lipid oxidation, water transpiration, and hydrolysis of pectin components—contribute to the loss of firmness in fruits and vegetables during storage. Figure 4 depicts the findings of the hardness of control and treated red chili. According to Figure 4, the longer the storage period, the lower the F_max_ value, although the edible coating treatment can mitigate the Firmness decline. One of the most significant quality characteristics of all fruits and vegetables, including red chili, is firmness. Tissue softening is therefore a serious problem that has an impact on visual quality. 

Based on Figure 4, the loss of firmness in coated chili fruits was reduced due to barrier qualities against gases and water loss transpiration, hence keeping cell turgor and firmness. During storage, firmness in all groups showed a downward trend, and firmness in the coated group of each variety was significantly higher than its control group, which can be explained by the coating reducing the respiration rate and ethylene production, thereby reducing the activity of cell-wall-degrading enzymes. The findings are consistent with prior research on the application, which looked at the influence of the edible coating on the firmness of fresh fruit and vegetables during storage. Previous research has also shown a similar result, in which the application of an edible coating of biopolymers influenced firmness through the application of an edible coating in cherry fruits during storage in order to reduce the firmness due to water loss transpiration [5]. In addition to the transpiration, this is a result of protopectin decomposing into soluble pectin under the action of cell wall degrading enzymes such as pectin galactosidase, polygalacturonase, and pectin methylesterase, which reduce adhesion between cells and the mechanical strength of the cell wall, leading to the softening of fruit tissues and decreased firmness [5].

### 2.5. Color 

#### 2.5.1. Lightness

Color and visual appearance are significant quality criteria that have a direct impact on the customer’s sense of quality. It is one of the most important and distinctive characteristics of red chili. The L* or lightness value on red chili samples that were treated with a coating treatment of a pectin–gelatin composite solution incorporated with garlic essential oil and samples that were not given any treatment (control) can be seen in Table 1. The brightness (L*) of samples treated with the coating and the control samples decreased with storage time. The lowest brightness value was in the control sample, namely 20.4 ± 0.12 on the 14th day of storage. This decrease in the brightness value is due to the activity of the PPO (polyphenol oxidase) enzyme which causes a decrease in brightness to browning in fruits. Browning reactions can occur due to the reaction of oxygen with polyphenolic compounds catalyzed by polyphenol oxidase enzymes to form brown melanin compounds. PPO (Polyphenol Oxidase) enzyme activity produces a loss in brightness on the surface of the fruit flesh and leads it to brown [21]. Oxygen can react directly with polyphenol compounds if some cells or tissues are exposed due to injury. Enzymatic browning degrades the aesthetic look of fresh/fresh-cut fruits and vegetables, induces undesired taste changes, and promotes nutritional loss. Such modifications make the goods less appealing to customers. In any case, browning damages the original color of the product [22]. However, the drop in brightness value is larger in the control sample than in the sample treated with the edible coating because the edible coating can minimize the interaction between oxygen and polyphenol chemicals. Gelatin–pectin combined with garlic essential oil as a component of edible coatings has high oxygen barrier properties [23,24].

The brightness of the samples treated with either 2% or 3% garlic essential oil did not have many different results (*p* > 0.05). In samples 5 and 6, the brightness value on the 14th day of storage is close to the brightness value of the control sample. The decrease in brightness in controls tends to be greater than that of samples treated with edible coatings. Therefore, it can be concluded that the provision of edible coatings can maintain better brightness due to the presence of a layer as a barrier that reduces contact between oxygen and polyphenolic compounds. Previous research also reported the same thing, namely that the brightness value (L) of bell pepper decreased significantly during the storage period at room temperature and cold temperature [32,33]. This could be explained as coating formulations being more permeable to O2 due to the presence of a plasticizer, which decreases the intermolecular interactions among adjacent polymeric chains and facilitates gas mobility and permeability, which are functions that could cause changes in color due to oxidation damages that will influence the brightness value. In addition, the main function of the effect of coatings and edible films is to preserve changes in physicochemical parameters and biochemical processes such as enzymatic oxidation caused by polyphenol oxidase (PPO), which is responsible for the browning. This is in contrast with previously published works indicating the effectiveness of coatings [34]. 

#### 2.5.2. a (Degree of Redness)

The results of the examination of the reddish (a*) color of red chili coated with an edible gelatin composite coating are shown in Table 1. According to Table 1, the red parameter (a) of all red chili samples decreased significantly (*p <* 0.05) during storage. Because some carotenoid pigments in red chili can function as antioxidants, it is sensitive to light and oxygen during storage [25]. Samples with coatings of gelatin–pectin composite incorporated with garlic essential oil tend to protect against the reduction of a value during storage. The decrease in the number suggests that coatings can keep color more effectively. This might be due to the altered environment in the fruit caused by the edible covering, which influences the respiration rate, postponing color difference among samples more than the storage duration itself [26].

On the 14th day, it was seen that the control sample had a lower redness value than the treated sample. A high redness value (a*) of chilies during storage indicates the red color of chilies is approaching bright red, and the lower a* value means that the red color of the chilies is closer to dark red. This change can be caused by the oxidation of carotene and xanthophyll pigments, which are sensitive and easily damaged if exposed to light and oxygen [1]. Treatment with an edible pectin–gelatin composite coating incorporated with garlic essential oil could maintain a better red color value in the sample during storage at room temperature because the respiration rate slowed down.

#### 2.5.3. b (Degree of Yellowness)

The results of the examination of the yellowish parameter (b*) on red chili covered with an edible coating of gelatin–pectin mixed with garlic essential oil are shown in Table 1. On the first day, the b* value ranged from 17–28; on the 14th day, it was seen that the control sample had a lower value than the treated sample. According to Table 1, the yellow parameter of all red chili samples tends to fade with time. The decrease in the b value also suggests that coatings can hold color more effectively. This can also be attributed to the protective effect of edible coating [8].

### 2.6. Titratable Acidity

**Figure 5:**
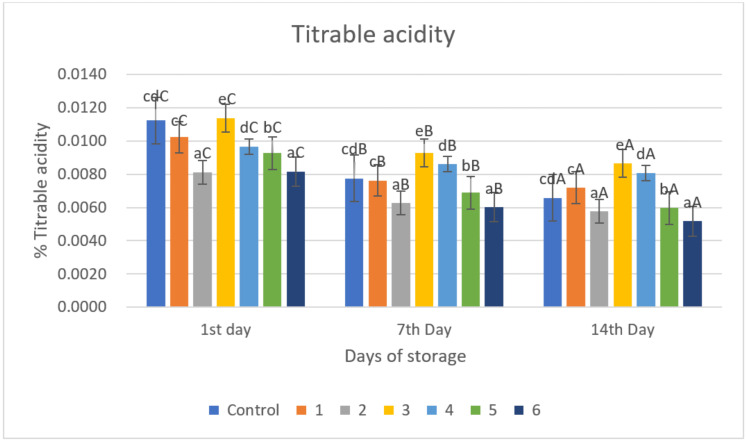
Titrable acidity of red chili during storage. ^a–e^ non-capital letters show the difference between the day of storage (*p* < 0.05). ^A–C^ capital letters show the difference between the day of storage (*p* < 0.05). Control: Uncoated Sample; 1: Gelatin75%-Pectin25%-Garlic Essential Oil2%; 2: Gelatin75%-Pectin25%-Garlic Essential Oil Oil3%; 3: Gelatin50%-Pectin50%-Garlic Essential Oil Oil2%; 4: Gelatin50%-Pectin50%-Garlic Essential Oil Oil3%; 5: Gelatin25%-Pectin75%-Garlic Essential Oil Oil2%; 6: Gelatin25%-Pectin75%-Garlic Essential Oil Oil3%.

Titratable acidity (TA) is a measure of the amount of acid in a solution and is believed to be an indicator of chili maturity [26,27]. It demonstrates that the titrable acidity of all samples declined as storage time increased. There is a significant difference (*p* < 0.05) in TA changes over the storage period (Figure 5). Throughout the storage time, the value of acidity (TA) decreased for all treatments. The decrease in TA in all treatments over the storage period is consistent with the usual process of fruit ripening. The coating treatment considerably (*p* < 0.05) increased the TA during storage durations. This might be because the coating treatment makes less O_2_ accessible for the respiratory process, thereby delaying the usage of organic acids. As a result, the decrease in TA during storage might be caused by a variety of reasons, including organic acid breakdown. Organic acids, such as citric acid, are important substrates for respiration. Acidity drop and pH increase are likely to occur in rapidly respiring fruits. External application or coating decreases respiration rates and inhibits organic acid activity. The gelatin–pectin composite with garlic essential oil treatment performed better in sustaining TA and pH at room temperature and under storage conditions than control samples, most likely due to its buffering function for organic acid component degradation. The findings are consistent with prior studies of edible coating for green chilies on the TA and pH of green chilies while they were stored [35]. The results showed that using a chitosan–pullulan composite edible film kept the acidity and pH of bell peppers stable throughout storage [33]. This might be related to the activation of defense enzymes slowing down the respiration rate, metabolic activity, and regulation of enzymatic activity and anthracnose.

TA values in all samples decreased significantly (*p* < 0.05) with increasing storage time (Figure 5). The biggest decrease in TA values was in the control sample, namely 0.0112, 0.0078, and 0.0066% on storage days 1, 7, and 14, respectively. The gelatin–pectin composite incorporated with garlic essential oil proved to be better at retaining TA under room temperature storage conditions (±29 °C, RH ± 69%) compared to the control sample. This study observed that TA contents of red chili in all groups showed a downward trend and that coating treatment inhibited the decomposition of TA due to the oxygen barrier effect of the coating reducing the oxygen concentration inside the fruit, which also reduced the intensity of respiration and related enzyme activities, thereby reducing the consumption of TA.

### 2.7. Total Soluble Solids

The total soluble solids (TSS) of fruits and vegetables are known to be an essential determinant in consumer acceptability. Because of the hydrolytic conversion of complex polysaccharides into simpler sugars and the transformation of pectic components, the TSS behavior of red chili steadily rises with the progression of time storage.

**Figure 6:**
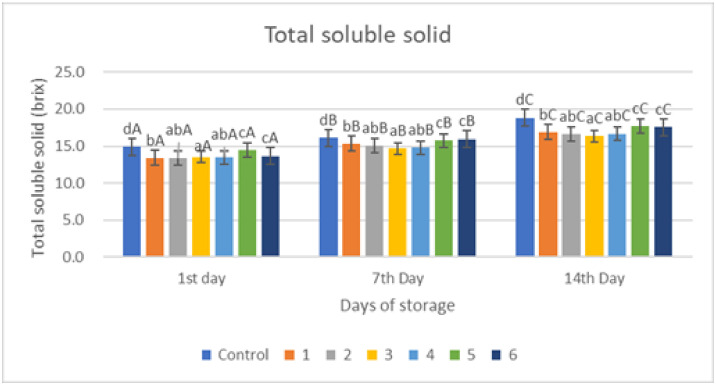
Total Soluble Solid of red chili during storage. ^a–d^ non-capital letters show the difference between the day of storage (*p* < 0.05). ^A–C^ capital letters show the difference between the day of storage (*p* < 0.05). Control: Uncoated Sample; 1: Gelatin75%-Pectin25%-Garlic Essential Oil2%; 2: Gelatin75%-Pectin25%-Garlic Essential Oil Oil3%; 3: Gelatin50%-Pectin50%-Garlic Essential Oil Oil2%; 4: Gelatin50%-Pectin50%-Garlic Essential Oil Oil3%; 5: Gelatin25%-Pectin75%-Garlic Essential Oil Oil2%; 6: Gelatin25%-Pectin75%-Garlic Essential Oil Oil3%.

The analysis of total soluble solids in red chilies coated with an edible coating of gelatin–pectin composite incorporated with garlic essential oil shown in Figure 6 can be taken as an example of the quantity of sugar in a material. Total soluble solids tend to increase during storage. The increase in the total value of soluble solids during storage is caused by the accumulation of glucose as a result of the hydrolysis of carbohydrates, which is faster than the process of converting glucose into energy and H_2_O [36]. During the ripening process, the carbohydrate content in red chili can also be hydrolyzed into glucose, fructose, and sucrose. Reducing sugar levels can change following the fruit respiration pattern [37]. The appearances of the coated and uncoated red chili samples are shown in Figure 6. The change of physicochemical characteristics including TSS will also give effect to the appearance of the red chili during storage.

The value of the increase in the total dissolved solids value of the control sample was significantly different (*p* < 0.005) compared to the treated sample. On the first day, the control sample had a value of 14.9 brix and increased to 18.8 brix on day 14 (Figure 6). There was a lower increase in value in the treated samples 1, 2, 3, and 4, which were around 16 brix on day 14. Samples 5 and 6 had higher brix values, namely 17.72 and 17.56 brix, and it can be seen that a decrease in gelatin content increased the total dissolved solids, which is because the gelatin layer is proven to be a good barrier thereby delaying the ripening process so that the total dissolved solids value becomes lower.

### 2.8. Antioxidant Activity

**Figure 7:**
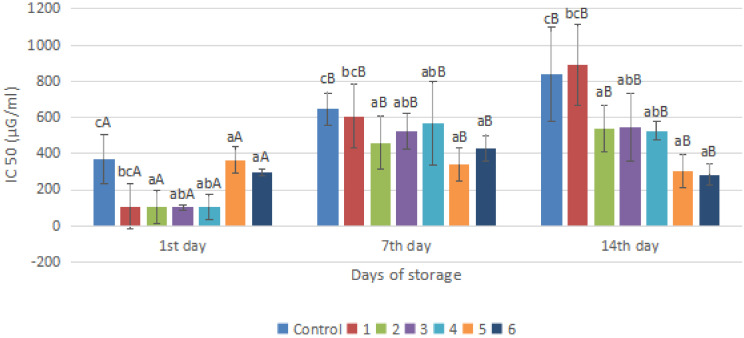
Total antioxidant of red chili during storage. ^a–c^ non-capital letters show the difference between the day of storage (*p* < 0.05). ^A, B^ capital letters show the difference between the day of storage (*p* < 0.05). Control: Uncoated Sample; 1: Gelatin75%-Pectin25%-Garlic Essential Oil2%; 2: Gelatin75%-Pectin25%-Garlic Essential Oil Oil3%; 3: Gelatin50%-Pectin50%-Garlic Essential Oil Oil2%; 4: Gelatin50%-Pectin50%-Garlic Essential Oil Oil3%; 5: Gelatin25%-Pectin75%-Garlic Essential Oil Oil2%; 6: Gelatin25%-Pectin75%-Garlic Essential Oil Oil3%.

Figure 7 depicts the total antioxidant (IC_50_) analysis of red chili covered with an edible coating of a gelatin–pectin composite supplemented with garlic essential oil. It can be seen in Figure 7 that there is a significant difference in the control sample compared to the treatment sample (*p* < 0.005), especially on the seventh day, but for all coating treatments on the same day, there is no significant difference. On the first day of storage, a low IC_50_ value was present at coating 1–coating 4; coating 5 and 6 were almost the same as the control, but were still below.

There was a significant increase in all treatments on day 7 and some coatings still increase in IC_50_ value until day 14 and some decreased. This fluctuating result is due to the level of maturity of chili itself; the growth conditions of the plant can be different, thus it will affect the antioxidant capacity of the fruit tissue. On the last day of storage, it was seen that the addition of pectin and garlic essential oil to the coating solution affected the IC_50_ value. The greater the concentration of pectin, the lower the IC_50_ value.

The addition of garlic oil will affect its antioxidant activity, and there was an increase in the percentage of inhibition along with the addition of the concentration of garlic oil used. This is because garlic contains the main compounds callicin, alliin, allyl cysteine, and allyl disulphide, which are active against free radical damage [38]. In Figure 7, it can be seen that the control sample has a high IC_50_ value compared to all coating samples on all observation days, which is in line with the results of vitamin C analysis in this study where coating samples had the highest levels of vitamin C compared to the control. Vitamin C has an important role in antioxidant capacity, and it is an organic acid needed by fruit as a substrate in the respiration process. The treatment given is expected to inhibit the respiration process so that the vitamin C content is maintained and, of course, it will also maintain the antioxidant capacity of the fruit [39].

Based on Figure 7, on the first day of storage, the lowest IC_50_ value was in samples 1 to 4 and the highest was in the control sample, which was 368.76. On the seventh day of storage, there was a significant increase in all treatments; later, almost all samples still experienced an increase in IC_50_ values until day 14, and some also decreased. This fluctuating yield is due to several factors such as the maturity level of the chili itself and different plant growth conditions that can affect the antioxidant capacity of the fruit tissue [40]. In general, on the 14th day, it was seen that the addition of pectin and garlic essential oil to the coating solution affected the IC_50_ value. The greater the pectin concentration, the lower the IC_50_ value, namely in samples 5 and 6. Among all the samples, the best was the one containing higher essential oils. The addition of garlic essential oil will thus affect its antioxidant activity. There was an increase in the percentage of inhibition along with the addition of the concentration of garlic essential oil used. This is because garlic contains the main compounds callicin, alliin, allyl cysteine, and allyl disulphide, which are active against damage caused by free radicals [27]. Coating chili peppers has been shown to provide a better IC_50_ value, and the total radical-scavenging activity of chili peppers is also influenced by the synergism between the total antioxidants in the sample, such as vitamins C and E, and the content of carotenoid pigments [41]. In this study, the treated samples were able to retain their vitamin C content better than the control, where vitamin C is an organic acid needed as a substrate in the respiration process. Coating can inhibit this process so that the vitamin C content is better maintained and, of course, it will also be able to maintain the antioxidant capacity of the fruit [13].

### 2.9. Decay Incidence

One of the causes of rotting in red chili besides anthracnose is the damage caused by putrefactive fungi such as *Aspergillus* sp. and *Fusarium* sp. The percentage of damage (decay incidence) can be seen in Table 2. A significant difference (*p* < 0.05) was seen in the damage between the control sample and the treated sample. On the first day, almost all of the treated samples were not damaged, while the control samples on the first day were damaged by 22.22%. Along with the storage time, the percentage of damage caused by these microorganisms increased, and on the 7th day, the treated samples began to experience damage. The lowest damage was in coated samples 6 and 3 and the highest was in the control sample. At the end of storage on the fourteenth day, the lowest damage was in sample 3 with a 50:50 composite composition and the highest damage was in the control sample of 68.89%. In general, the data shows that the addition of more garlic essential oil (3%) can slightly reduce the damage caused by these microorganisms. This may be due to the addition of 2% essential oil which is good enough to prevent damage caused by microorganisms in red chili. However, the addition of essential oils to the coating solution still shows significant potential for antimicrobial activity, and the addition of essential oil to chitosan can extend the shelf life by inhibiting the growth of unwanted microorganisms [9,42].

**Figure 8:**
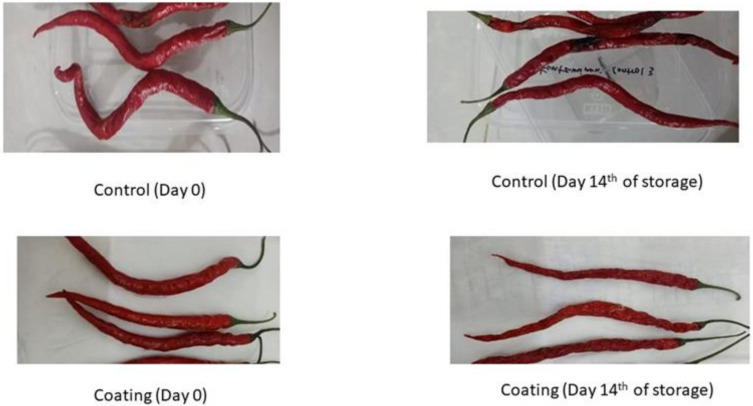
Appearance of Red Chili.

Furthermore, the coating helps delay senescence, which makes the commodity more susceptible to pathogen infection as a result of loss of cellular or tissue integrity. A similar study was also conducted on bell peppers, in which the fruit coated with chitosan and chitosan-gelatin composite significantly inhibited microbial spoilage, namely 7.4% and 10.6%. This value is two to three times smaller than that of the uncoated peppers, which is 25.3%. The addition of gelatin to the coating does not reduce the antimicrobial effect [32]. The appearance of red chili can be seen in Figure 8.

## 3. Conclusions

The treatment of edible coatings from gelatin–pectin composites, especially the concentration of 50:50, is incorporated with garlic essential oil with a concentration of 2% (coating 3) on red chili commodities that are stored for 14 days at room temperature, which can provide a protective effect against changes in several physicochemical properties such as inhibition of weight loss by 36.36% and maintaining the decay incidence that began on the 7th day as 17.78% to 28.89% on the 14th day of storage. The edible coating treatment of the gelatin–pectin composite, especially the concentration of 50:50 that was incorporated with garlic essential oil with a concentration of 2% (coating 3), had a hardness value of 0.547 kg/84 mm^2^, maintained acidity of 0.0087%, maintained vitamin C content of 2.237 mg/g, antioxidant activity (IC_50_) 546.587, and provided a protective effect on red chili discoloration to maintain a better value of total dissolved solids. Our findings reveal that the application of the edible coating to red chilies can be an effective method to protect the quality of red chilies and has the potential to extend its storage life up to the 14th day at room temperature storage (±29 °C, RH ± 69%).

## 4. Materials and Methods

### 4.1. Materials

Red chili *(C. annum* L.) aged 1–2 days of harvesting (Pasar Jatimulyo, Lampung, Indonesia), garlic essential oil (Lansida group, Yogyakarta, Indonesia), tilapia fish gelatin (Redman fish gelatin, Ang Mo Kio, Singapore), Low Methoxyl Pectin (LMP) (Campectin 4510, Madrid, Spain), and other chemical reagents. 

### 4.2. Preparation of Edible Coatings

First, the dissolving gelatin and pectin in the distilled water are tested separately with a concentration of 3% (*w*/*v*) at 50 °C for 120 min. Afterward, several formulas of the gelatin and pectin solutions with gelatin:pectin ratios of 75:25, 50:50, and 25:75 (*v*/*v*) were then homogenized by stirring using a magnetic stirrer at 40 °C for 3 h. Afterward, 7% glycerol was added as a plasticizer and stirred at 40 °C for 60 min. Garlic oil was added to the solution according to the concentration variations of 2 and 3% (*w*/*v*) and then added between 20 (15% *v*/*v*) while stirring continued for 90 min. The edible coating solution was then ready to use.

### 4.3. Application for Coating on Red Chili

**Figure 9:**
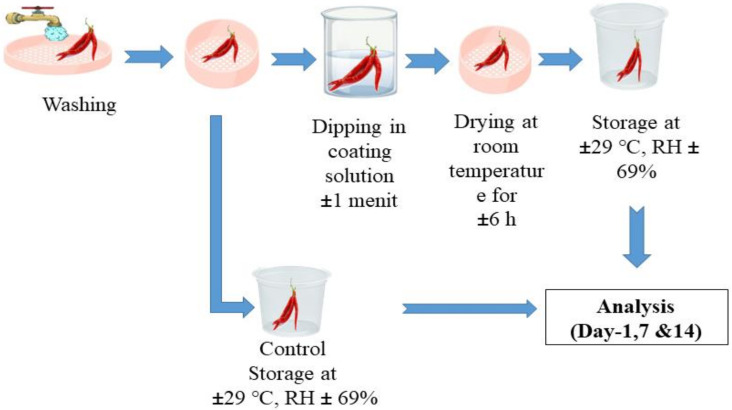
Application of edible coating on red chili.

The process of coating the edible coating solution on red chili (*Capsicum annum* L.) referring to the research of Bermudez-Oria et al. (2017) was carried out using the dipping technique. First, the red chili samples were dipped in the coating solution for 1 min, then drained and dried at room temperature, and then each sample was stored at room temperature for 14 days and analyzed on days 1, 7, and 14. The evaluation of the sample was carried out including physical analysis (weight loss, firmness, and color) and chemical analysis (titratable acidity, vitamin C content, and total soluble solids). The application for coating the red chili can be seen in Figure 9.

### 4.4. FTIR

The coating solution was applied to the KBr plate. Then, the plate was loaded in FTIR spectroscopy (Thermo, Japan) with a scan range from 400 to 4000 cm^−1^.

### 4.5. Weight Loss

The calculation of weight loss is presented below [42].

$$\frac{\mathrm{Weight}\;\mathrm{loss}\;\left(\%\right)=\mathrm{initial}\;\mathrm{weight}\left(g\right)\;-\;\mathrm{final}\;\mathrm{weight}\;\left(g\right)\;\text{×}\;100\%}{\mathrm{Initial}\;\mathrm{weight}\;(g)}$$

### 4.6. Total Vitamin C

Analysis of vitamin C was carried out using modified iodine titration [43]. An amount of 2.5 g of homogenized red chili sample was weighed and put in a 100 mL volumetric flask, then 100 mL of distilled water was added into the flask. Afterward, the solution was filtered to separate the filtrate. The obtained filtrate was placed in an Erlenmeyer flask, then 2 mL of 1% starch solution was added. Afterward, titration was performed using 0.01 N Iodine solution. Vitamin C content was calculated as mg ascorbic acid/g sample (1 mL 0.01 N Iodine = 0.88 mg ascorbic acid).

### 4.7. Color

The Minolta CR-400 Chromameter was used to analyze color intensity. The sample is placed on top of the chromameter sensor, then light is fired at the part to be measured so that the values of L (lightness), a (green-red chromaticity), and b (yellow-blue chromaticity) will appear on the chromameter display.

### 4.8. Firmness

Firmness measurements were carried out using a Fruit hardness tester KM-1 (Fujiwara, Tokyo, Japan). The firmness test was measured based on the level of resistance of the fruit to the rheometer needle. The maximum load is 1 kg. Each measurement was repeated three times per test sample. The value of fruit firmness is read on a pointer scale in kg/84 mm^2^ units. This value indicates the compressive force required by the needle to pierce the fruit sample.

### 4.9. Titratable Acidity (TA)

TA was calculated based on the AOAC method which was expressed in grams of acid/100 g of the product. The sample was titrated using 0.1 mol/L NaOH and phenolphthalein as an indicator.

### 4.10. Total Soluble Solid (TSS)

The Atago Master-53M refractometer was used to measure total soluble solids. One to two drops of the sample chili extract are put on the prism of the refractometer at room temperature, and the Brix % is read through the refractometer’s eyepiece.

### 4.11. Antioxidant Activity

The DPPH technique was used to assess antioxidant activity (2,2-diphenyl-1-picrihydrazil). A UV-Vis spectrophotometer with a wavelength of 517 nm was used to measure the absorption. The antioxidant activity of the sample is expressed as a percentage of radical scavenging activity, or the sample’s capacity to trap free radical molecules. The greater the number of free radical molecules that can be collected, the greater the antioxidant activity content of the sample. The mathematical results are used to create regression curves. The IC50 concentration is calculated using the resulting linear regression equation.

$$\mathrm{DPPH}\;\mathrm{radical}\;\mathrm{scavenging}\;\mathrm{activity}\;\left(\%\right)=\frac{A\;\mathrm{blanko}\;-\;A\;\mathrm{sample}}{A\;\mathrm{blanko}}\;\times \;100\%$$

### 4.12. Decay Incidence

The percentage of decay incidence of coated and uncoated (control samples) red chili were analyzed on days 1, 7, and 14 at room temperature storage (±29 °C, RH ± 69%). The decay incidence was determined by observing whether or not decay was visible on the sample surface, then the percentage of damaged samples was calculated. Samples are considered damaged if there is fungal mycelium on the surface and decay occurs. The results of these observations are expressed as the percentage of samples contaminated with fungal mycelium.

$$\mathrm{Decay}\;\mathrm{incidence}\;\left(\%\right)=\frac{\mathrm{Amount}\;\mathrm{of}\;\mathrm{contaminated}\;\mathrm{sample}}{\mathrm{Total}\;\mathrm{sample}}\;\times \;100\%$$

### 4.13. Statistical Analysis

SPSS 25 was used for the statistical analysis. The analysis of variance (ANOVA) and DMRT were performed with a degree of confidence of 95%. The studies were carried out in at least triplicate, and the results were given as mean standard deviation.

## Figures and Tables

**Table 1:** Color Parameter during Storage.

Sample	Color Parameter								
	L*			a*			b*		
	1st Day	7th Day	14th Day	1st Day	7th Day	14th Day	1st Day	7th Day	14th Day
Control	27,42 ± 1.16	24.43 ^Aa^ ± 0.24	20.4 ^Aa^ ± 0.12	38.94 ^Bb^ ± 1.14	30.38 ^Aa^ ± 1.33	28.02 ^Aa^ ± 0.53	24.69 ^BCb^ ± 1.94	15.71 ^Aa^ ± 0.80	14.59 ^Aa^ ± 0.89
1	28.22 ± 0.57	25.87 ^Bb^ ± 1.07	25.42 ^Cb^ ± 1.27	34.77 ^Aa^ ± 1.61	32.83 ^Ba^ ± 4.06	30.41 ^Ba^ ± 3.73	21.70 ^Ba^ ± 0.86	19.71 ^Ba^ ± 3.78	16.71 ^Ba^ ± 4.39
2	28.16 ± 0.60	25.99 ^Bb^ ± 0.53	24.29 ^Cb^ ± 0.72	37.93 ^Ab^ ± 1.62	38.50 ^Bb^ ± 1.15	35.70 ^Bb^ ± 1.19	25.84 ^Bb^ ± 1.86	25.84 ^Bb^ ± 0.98	24.48 ^Bb^ ± 1.34
3	26.84 ± 1.00	26.46 ^Bb^ ± 1.16	26.11 ^Db^ ± 1.38	33.68 ^Aa^ ± 1.20	32.69 ^Ba^ ± 2.60	31.34 ^Ba^ ± 3.09	17.91 A^a^ ± 2.16	17.87 ^Ba^ ± 3.21	18.62 ^Ba^ ± 1.23
4	28.49 ± 0.57	27.48 ^Bb^ ± 0.89	27.54 ^Db^ ± 0.80	39.47 ^Ab^ ± 1.10	37.74 ^Ab^ ± 2.58	33.63 ^Bb^ ± 1.29	24.18 A^b^ ± 0.72	25.22 ^Bb^ ± 2.59	20.60 ^Bb^ ± 0.30
5	28.37 ± 0.66	25.50 ^Bb^ ± 0.26	22.20 ^Bb^ ± 0.95	39.73 ^Ba^ ± 1.30	31.20 ^Ba^ ± 2.21	28.63 ^Ba^ ± 0.90	25.92 ^Ca^ ± 2.41	17.94 ^Ba^ ± 1.55	15.44 ^Ba^ ± 1.29
6	29.40 ± 0.87	26.08 ^Bb^ ± 0.73	23.50 ^Bb^ ± 1.28	39.74 ^Bb^ ± 1.70	38.92 ^Bb^ ± 2.45	33.73 ^Bb^ ± 1.17	28.08 C^b^ ± 2.20	27.82 ^Bb^ ± 2.83	26.68 ^Bb^ ± 5.10

^a, b^ non-capital letter show the difference between the sample (*p* < 0.05). ^A–C^ capital letter show the difference between the day of storage (*p* < 0.05). Control: Uncoated Sample; 1: Gelatin75%-Pectin25%-Garlic Essential Oil2%; 2: Gelatin75%-Pectin25%-Garlic Essential Oil Oil3%; 3: Gelatin50%-Pectin50%-Garlic Essential Oil Oil2%; 4: Gelatin50%-Pectin50%-Garlic Essential Oil Oil3%; 5: Gelatin25%-Pectin75%-Garlic Essential Oil Oil2%; 6: Gelatin25%-Pectin75%-Garlic Essential Oil Oil3%.

**Table 2:** Decay incidence of red chilli during storage.

Sample	Decay Incidence (%)		
	1st Day	7th Day	14th Day
Control	22.22 ^cA^ ± 12.02	42.22 ^cB^ ± 6.67	68.89 ^cC^ ± 14.53
1	0.00 ^bA^ ± 0.00	33.33 ^bB^ ± 10.00	48.89 ^bC^ ± 10.54
2	0.00 ^abA^ ± 0.00	22.22 ^abB^ ±12.02	40.00 ^abC^ ± 10.00
3	2.22 ^aA^ ± 6.67	17.78 ^aB^ ± 15.63	28.89 ^aC^ ± 14.53
4	0.00 ^bA^ ± 0.00	35.56 bB ± 13.33	42.22 bC ± 6.67
5	0.00 ^bA^ ± 0.00	33.33 ^bB^ ± 12.02	44.44 ^bC^ ± 18.56
6	4.44 ^aA^ ±8.82	11.11 ^aB^ ± 8.82	33.33 ^aC^ ± 16.67

^a–c^ non-capital letter show the difference between the sample (*p* < 0.05). ^A–C^ capital letter show the difference between the day of storage (*p* < 0.05). Control: Uncoated Sample; 1: Gelatin75%-Pectin25%-Garlic Essential Oil2%; 2: Gelatin75%-Pectin25%-Garlic Essential Oil Oil3%; 3: Gelatin50%-Pectin50%-Garlic Essential Oil Oil2%; 4: Gelatin50%-Pectin50%-Garlic Essential Oil Oil3%; 5: Gelatin25%-Pectin75%-Garlic Essential Oil Oil2%; 6: Gelatin25%-Pectin75%-Garlic Essential Oil Oil3%.
